# Plasma Treatment of Nanocellulose to Improve the Surface Properties

**DOI:** 10.3390/polym16172516

**Published:** 2024-09-04

**Authors:** Gregor Primc, Miran Mozetič

**Affiliations:** Department of Surface Engineering, Jozef Stefan Institute, Jamova cesta 39, 1000 Ljubljana, Slovenia; gregor.primc@ijs.si

**Keywords:** nanocellulose, surface, wettability, plasma, upscaling

## Abstract

Nanocellulose is among the most promising materials for enhancing the mechanical properties of polymer composites. Broad application is, however, limited by inadequate surface properties. A standard technique for tailoring the surface composition and wettability of polymers is a brief treatment with non-equilibrium gaseous plasma, but it often fails when treating materials with a large surface-to-mass ratio, such as cellulose nanofibers. In this paper, the theoretical limitations are explained, the approaches reported by different groups are reviewed, and the results are interpreted. The treatment of dry nanocellulose is limited by the ability of uniform treatment, whereas the plasma treatment of nanocellulose dispersed in liquids is a slow process. The methods for enhancing the treatment efficiency for both dry and water-dispersed nanocellulose are explained.

## 1. Introduction

**Figure 1:**
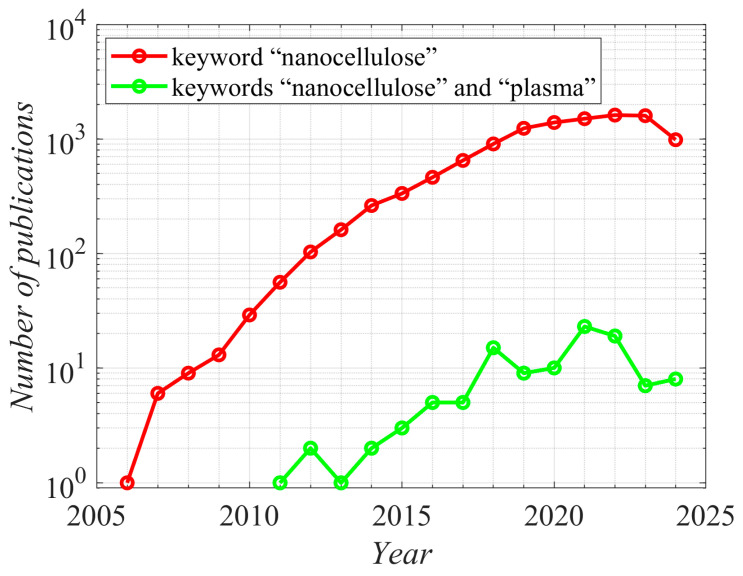
The number of scientific articles published annually searched for in the Web of Science with two different search strings. The y-axis is logarithmic. Source: Web of Science, retrieved on 20 August 2024.

Nanocellulose has attracted significant attention from the scientific community because of its promising applications, from aerogels [1] and bio-based composites [2] to advanced batteries [3]. The scientific literature on nanocellulose is vast. Searching Web of Science for the keyword “nanocellulose” gave well over 10,000 hits. Interestingly enough, only about 100 scientific articles were found by searching for “nanocellulose” and “plasma”. The first report about nanocellulose dates back to 2006, and the first report, which also mentions plasma, was published in 2011. The number of papers published annually is plotted in Figure 1.

Cellulose is the most abundant natural polymer, so any product synthesized from nanocellulose is regarded as ecologically friendly. Nanocellulose can be synthesized either in the form of nanofibers of a large aspect ratio [4] or nanocrystals [5]. Nanocellulose is a highly hydrophilic material, which is attributed to its surface hydroxyl groups [6]. Furthermore, the rich morphology and porosity of nanocellulose cause rapid soaking of polar liquids like water due to the capillary drag forces, so pure nanocellulose powder is often super-hydrophilic, i.e., a water droplet is immediately soaked after the deposition, and the water contact angle (WCA) cannot be determined unless the thickness of the nanocellulose film is so small that the substrate’s hydrophilicity/hydrophobicity plays a role [7]. In practical cases, however, the surface impurities prevent the super-hydrophilic character, and the nanocellulose surface should be oxidized to benefit from optimal dispersion [8]. High wettability is advantageous in many applications, such as in additives in the food industry [9], but it may also be disadvantageous in other applications like food packaging [10]. Therefore, the surface properties of the nanocellulose should be modified in order to achieve the desired properties of products made from nanofibers, nanocrystals, or both [11].

A standard method for surface modification of polymer materials is the application of non-equilibrium low-temperature gaseous plasma [12]. Plasma could be used either for surface functionalization of polymeric materials [13], etching [14], deposition of a thin film [15], or a combination of these effects [16]. Gaseous plasma is a source of charged particles (free electrons, positively and negatively charged ions), molecular radicals (free neutral atoms or fragments of molecular gases), and radiation. The intensity of the visible radiation is often marginal compared to the intensity of vacuum ultraviolet (VUV) radiation [17]. The effect of neutral radicals is limited to the very thin surface film, and the same applies to positively charged ions as long as the treated polymer is at the floating potential during the plasma treatment. The radiation will typically penetrate deep into the polymer materials, thus triggering polymer modification in the bulk material [18]. In fact, the effect of VUV radiation on polymer degradation has been known for decades [19] and used for relatively stable polymer hydrophilization [20]. Otherwise, all plasma-activated polymers are prone to hydrophobic recovery, i.e., the loss of surface wettability during storage [21].

Surface functionalization is among the first results of polymer treatment when using reactive gaseous plasma. Detailed analysis of the evolution of surface functional groups on very smooth polymers revealed that the surface of polyolefin is saturated with a layer of hydroxyl groups even after receiving the dose of neutral oxygen atoms below 10^21^ m^−2^ [22], and the treatment with larger doses causes etching [23]. Since a typical flux of O atoms from oxygen plasma is often in the order of 10^23^ m^−2^ s^−1^ [24], the surface is saturated with the polar functional groups in a fraction of a second. The saturation of the polymer surface with specific functional groups does not necessarily lead to optimal hydrophilicity because the surface groups are unstable, and hydrophobic recovery occurs. Some polymers exhibit complete hydrophilization only after receiving a dose of O atoms in the order of 10^23^ m^−2^ [25]. The doses for saturation of cellulose samples with specific functional groups are yet to be measured.

Plasma treatment is an established method for uniformly modifying the surface properties of polymers and polymer composites with a rather small surface-to-volume ratio. A scientific and technological challenge, however, is the treatment of powder materials. A comprehensive review of methods for treating polymer powders was published [26]. Briefly, the methods include stirring polymer powder in a dish, rotation of the discharge chamber, vibration of the conveyor belt, and fluidization. None of these methods is particularly useful for treating powders whose dimensions are below a micrometer, such as nanocellulose. Namely, the substantial surface-to-mass ratio of the nanocellulose favors some effects that could be otherwise neglected.

## 2. Theoretical Obstacles

The first consideration when studying the plasma treatment of nano-powder is the huge surface-to-mass ratio. The surface area of a nanofiber with the diameter *d* and the length *l* is in the approximation of long fibers (*l* >> *d*):

*A*_0_ = *N* π *d l*. (1)

Here, *N* is the number of fibers in the powder to be treated. The number of fibers with a single-fiber volume *V* in the nanocellulose powder of mass *m* is as follows:

*N* = *m*/(*V ρ*) = 4 *m*/(π *d* 2 *l ρ*), (2)

where *ρ* is the cellulose density (about 1.5 × 10^3^ kg m^−3^). Suppose the fiber diameter *d* is 10 nm and the length *l* is 100 nm. Equation (2) reveals that a mass of 1 kg will contain as many as *N* = 10^20^ fibers. The surface of the fibers in a mass of *m* is calculated from Equations (1) and (2) as follows:

*A* = (4 *m* π *d l*)/(π *d* 2 *l ρ*) = (4 *m*)/(*d ρ*). (3)

Considering the mass *m* of 1 kg, fiber diameter *d* of 10 nm, and the density *ρ* of 1.5 × 10^3^ kg m^−3^, the area of nanofibers in 1 kg of nanocellulose powder will be in the order of 10^5^ m^2^.

Suppose the surface area of the sample holder in a plasma reactor for the treatment of nanocellulose is *S*, and the available flux of plasma radicals onto this surface area is *j*. Let the nanofibers be uniformly distributed on the surface *S* and optimally stirred so that all nanofibers receive the same dose of plasma radicals. The time needed for uniform treatment of the nanofibers of the total area *A* with the dose of radicals *D* is as follows:

*t* = (*A D*)/(*S j*) = (4 *m D*)/(*d ρ S j*). (4)

Equation (4) reveals that the required treatment time is inversely proportional to the fiber diameter (*d*). The dose of radicals (*D*) that enables complete change in the surface wettability of many polymers is roughly 10^24^ m^−2^ [25]. Suppose the available surface of the plasma reactor (*S*) is 1 m^2^. The maximal available flux of plasma radicals (*j*) in weakly ionized plasma beneficial for surface functionalization of polymer materials is close to 10^23^ m^−2^ s^−1^ [27]. If the stirring of the nanocellulose powder with a mass of 1 kg consisting of long nanofibers with a diameter of 10 nm is optimal, the complete wettability of the fibers will be accomplished in the following time:

*t* = (4 kg 10^24^ m^−2^)/(10 nm 1.5 × 10^3^ kg m^−3^ 1 m^2^ 10^23^ m^−2^ s^−1^) = 2.666667 × 10^6^ s = 740.74 h = 30.86 days (5)

Equations (1)–(5) reveal that the treatment time will be prohibitively long, even in the case of optimal stirring and maximal flux of plasma radicals. The optimal conditions are challenging to achieve, so the treatment time will be even longer in practical cases, especially when the flux of plasma radicals is inadequate [28]. Equation (4) reveals that the treatment time could be shorter if reactors of a large surface area (*S*) of the sample holder are used. However, in Equation (5), we took into account the plasma reactor area (*S*) of 1 m^2^, which is already quite large. The required dose (*D*) depends on the desired surface finish and may be below the value needed for a complete change in the surface wettability, i.e., 10^24^ m^−2^, especially when condensable radicals are deposited onto the polymer surface [29].

**Figure 2:**
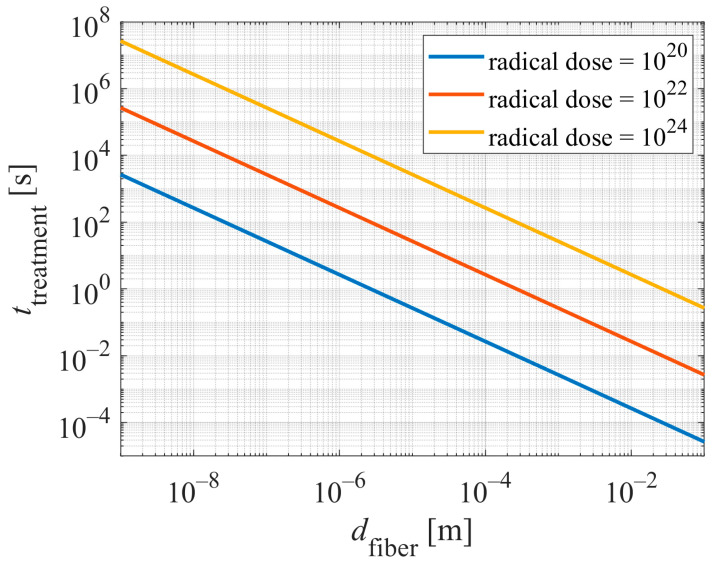
The required treatment time for complete modification of the surface properties of cellulose nanofibers versus the fiber diameter. The values are calculated for the required doses of 10^20^, 10^22^, and 10^24^ m^−2^ of plasma radicals and a total nanofiber mass of 1 kg.

The treatment time becomes reasonable only for fibers of larger diameter. Figure 2 represents a plot of the required treatment time versus the diameter of long fibers (in the approximation *d* << *l*). The parameter is the required dose (*D*), and the values are calculated for the flux of plasma radicals (*j*) of 1×10^23^ m^−2^ s^−1^ and the surface area of the sample holder in the plasma reactor (*S*) of 1 m^2^.

Figure 2 reveals the difficulties arising from the simple fact that cellulose nanofibers’ (CNFs) diameter is around 10 nm. Larger fibers, for example, those with a dimension of several micrometers, will be functionalized in a reasonable time, i.e., between less than 0.01 s and 10, depending on the radical dose. Depending on the required dose of plasma radicals, the mass of 1 kg fibers of diameter 10 µm may be uniformly treated within an hour. Commercial reactors for treating polymer powders enable reasonable modification of the surface wettability within less than an hour for batches of about 1 kg [30]. The required treatment time becomes adequate (a minute or below) for macroscopic objects (fiber diameter over 1 mm in Figure 2). The treatment time of polymer foils and similar objects made from cellulose, as reported by different authors, is actually in the range between a few seconds and several minutes [11].

## 3. Literature Survey on Plasma Treatment of Nanocellulose

Considering the theoretical obstacles summarized in Section 2, it is not surprising that very few teams have tackled the modification of nanocellulose through gaseous plasma treatment. Furthermore, the stirring or mixing of nano-powder is not trivial, so the macroscopic quantities of nanocellulose were treated by a few teams only. Below is a brief literature survey with the major results reported by different teams. The results are explained by taking into account the specifics of plasma sustained by various discharges as well as the peculiarities of methods for surface characterization.

### 3.1. Treatment of Dry Powder

**Figure 3:**
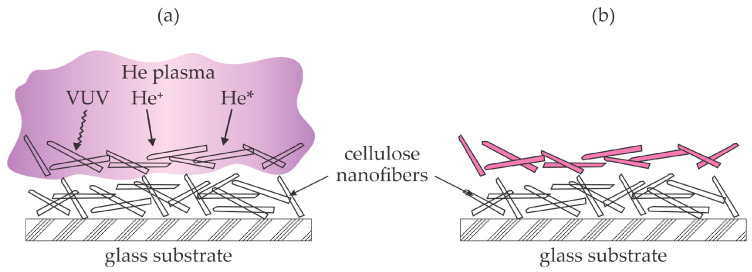
Illustration of plasma treatment of dry nanocellulose fibers using atmospheric pressure plasma sustained by high-impedance discharge in a noble gas. (**a**) The uppermost fibers are treated by plasma radicals; (**b**) only the uppermost fibers are modified if no stirring is employed.

Kusano et al. provided one of the first reports on the plasma treatment of nanocellulose [8]. The authors deposited a water suspension of cellulose nanofibers on glass substrates of a surface of about 20 cm^2^. The suspension was thoroughly dried, and a dry film of nanocellulose of an approximate thickness of 7 µm was exposed to helium plasma sustained by a dielectric barrier discharge at atmospheric pressure for half a minute. The authors reported that the discharge power was as large as 100 W. The plasma treatment caused an increased oxygen concentration, as revealed by X-ray photoelectron spectroscopy (XPS). The oxygen–carbon (O/C) ratio for untreated nanocellulose was 0.37, and it increased to 0.43 after treating it for half a minute at the discharge power of 100 W. High-resolution C1s spectra revealed a significant decrease in the concentration of C–C or C–H bonds from 36 to 27%. Interestingly enough, the concentration of the C–O bonds (the primary bonds in pure cellulose) also decreased from 48 to 44%, but the concentration of the carbonyl groups increased from 16 to 21%. The COO bonds (often ascribed to carboxyl groups) appeared in the surface layer of deposited nanocellulose with the thickness as probed by X-ray photoelectron spectroscopy (several nm), and their concentration was as large as 8%. The water contact angle (WCA) on untreated cellulose nanofibers was about 25° and decreased to about 15° after treating the samples at different discharge powers between 20 and 100 W. Both WCA and XPS revealed that the helium plasma treatment causes the formation of highly polar oxygen-rich functional groups on the surface of the film of dried nanofibers and, thus, increased hydrophilicity. On the other hand, no significant differences could be discerned among the attenuated total reflectance Fourier-transform infrared spectroscopy (ATR-FTIR) spectra of CNFs before and after the plasma treatment. The discrepancy between WCA/XPS and ATR-FTIR can be explained by the different information depths typical of these techniques. Namely, the ATR-FTIR probes the films of thickness several micrometers, while WCA and XPS are very surface sensitive with an information depth of up to several nm. The approach reported by Kusano et al. [8] is illustrated in Figure 3. Figure 3a shows a film of dense cellulose nanofibers. Helium plasma is a source of ions, metastables, and VUV radiation, which break bonds in the uppermost nanofibers. Minute quantities of water vapor and perhaps some oxygen from effluent gas cause oxidation of the nanofibers on the surface, but the nanofibers deep in the deposited film remain intact, as revealed from the ATR-FTIR results. The authors did not mix the powder deposited onto the substrates, so it is likely that shaded nanofibers received just a minimal dose of plasma radicals. The selected treatment time did not enable the required dose of reactive plasma particles to enable uniform modification of all deposited nanofibers, as explained in Section 2 and shown in Figure 2. Still, this is probably the only report on plasma functionalization of self-standing dry nanocellulose fibers.

### 3.2. Treatment of Dry Nanocellulose Products

**Figure 4:**
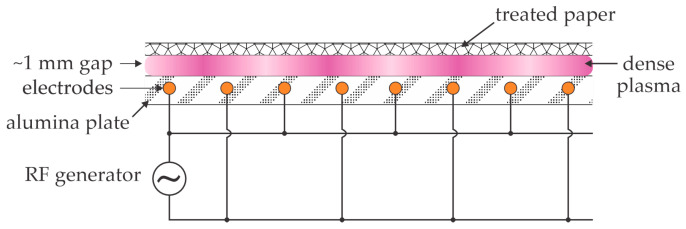
Schematic of the setup for treating nanocellulose paper and similar materials with plasma sustained by coplanar atmospheric pressure discharge at a power density as large as 100 W cm^−3^.

Vida et al. [31] used plasma sustained by coplanar dielectric barrier discharge to treat paper-like nanocellulose films. Plasma was sustained in the air at atmospheric pressure, and the treatment times varied between 1 and 16 s. The wettability was determined from the contact angle of a water droplet, and the chemical composition was evaluated by XPS. An excellent correlation between WCA and XPS was reported. The oxygen concentration on the surface of untreated nanocellulose paper was 34 at.% and increased to 42 at.% even after a second of plasma treatment. Further treatment did not cause a statistically significant composition modification as probed by XPS. The WCA on untreated nanocellulose paper was 72°, dropped to about 20° after a second of plasma treatment, and remained unchanged after that. High-resolution C1s XPS peaks showed a significant decrease in the intensity of the C–C peak already after 1 s of plasma treatment, which was attributed to the breaking bonds in the surface film of nanocellulose paper and oxidation of the unoxidized carbon bonds and perhaps also to the removal and/or oxidation of surface contamination. No attempt to deconvolute the high-resolution C1s peaks was reported by Vida et al. [31], probably because no statistically significant deviation of the peaks could be extracted from the acquired C1s peaks. The rapid saturation in both the chemical composition (as probed by XPS) and wettability (probed by WCA) is explained by the fact that the paper was relatively smooth (as compared to free-standing nanofibers), and the flux of plasma radicals was large because of the high power density of coplanar discharges. The authors reported a power density as large as 100 W cm^−3^. The schematic of the experimental setup useful for the rapid hydrophilization of nanocellulose paper, as adopted by Vida et al. [27], is shown in Figure 4.

Dimic-Misic et al. [32] used nitrogen plasma to improve the wettability of micro/nano-fibrillated cellulose films. The films were formed after drying the water suspension of the fibrillated cellulose using a sheet-former according to a modified ISO standard 5269-1 [33]. The water suspension was treated by enzymes for various treatment times, up to 5 h. The dry films were treated with plasma sustained by a dielectric barrier discharge (DBD) with a voltage of 6 kV in a pulsed mode with a pulsed frequency of 300 Hz. The plasma treatment time was half a minute or one minute. The XPS survey spectra showed carbon content between 10 and 30 at.%, and the authors determined the nitrogen content from as low as 0.02 at.% to about 3 at.%. The composition varied between particular samples treated by enzymes, but no evident trend was deduced. The polar component of the surface free energy remained within the limits of the experimental error for enzyme-treated times up to about 2 h and was between 60 and 70 mJ/m^2^ for plasma-treated samples, but a gradual decrease was observed for samples not treated by nitrogen plasma. The observations were attributed to the interaction of nitrogen plasma with the amorphous cellulose component in the non-hydrolyzed fibrils. The WCA was about 90° for untreated films and dropped to about 60 and 40° for films treated with nitrogen plasma for 30 and 60 s, respectively.

In another paper [34], the same team studied the influence of oxygen and nitrogen plasma treatment of the same material. The experimental system was the same as in [32]. When treating the films with oxygen or nitrogen plasma, the oxygen concentration, as deduced from XPS survey spectra, increased from approx. 30 to 40 at.%, whereas the nitrogen concentration remained at the XPS detection limit. The results reported in [32] and [34] show that nitrogen plasma does not enable significant functionalization with nitrogen but may lead to chemical degradation of the surface film. The authors mentioned that pulsed plasma was used to prevent overheating. Unfortunately, the discharge power was not mentioned in these scientific articles.

**Figure 5:**
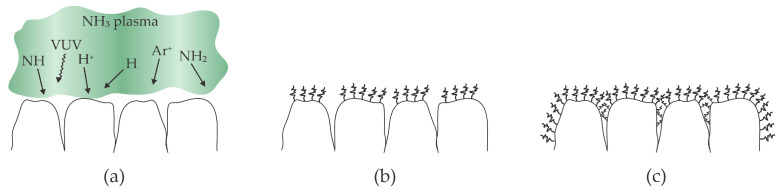
Surface reactions on the film made of cellulose nanocrystals upon exposure to plasma sustained in Ar with NH_3_ admixture. (**a**) Plasma is a source of positively charged ions, radicals, and VUV radiation; (**b**) short treatment time causes functionalization of exposed surface only; (**c**) prolonged treatment also causes functionalization in gaps and thus optimal wettability.

Matouk et al. [35] used plasmas sustained by DBD at atmospheric pressure in argon with an admixture of various hydrogen-containing gases (ammonia, methane, and silane) to tailor cellulose nanocrystal films’ surface properties. The discharge voltage depended on the gas mixture and was between 6 and 11 kV. The frequency of the sinusoidal power supply was about 5 kHz. The sonicated water suspension containing 2.7 wt.% cellulose nanocrystals was poured into Petri dishes and dried at ambient conditions. The WCA for untreated samples was 53°. XPS revealed the C and O concentrations for as-deposited films of 61 and 38 at.%, respectively. When ammonia was added to argon plasma, the WCA dropped to 25° after a minute of plasma treatment and continued decreasing with treatment time, reaching almost super-hydrophilic surface finish (WCA 8°) after one hour of plasma treatment. The XPS survey spectra showed about 10 at.% nitrogen, somewhat independent from the plasma treatment time. The concentrations of both C and O did not depend much on the treatment time and dropped to about 50 and 33 at.%, respectively. The rest were impurities such as Na and S. The discrepancy between the WCA and XPS measurements might be explained by the penetration of the plasma radicals, in particular N atoms deeper in the gaps between neighboring nanocrystals, enabling the surface functionalization with amide groups, which caused capillary drag forces and thus an almost super-hydrophilic surface finish after an hour of plasma treatment. When methane or silane was added to argon, the cellulose became hydrophobic, which was explained by the deposition of a thin film containing hydrocarbon and silica nanoparticles, respectively. The effects of treatment with plasma sustained in argon with an admixture of ammonia are illustrated in Figure 5.

Kutova et al. [36] treated bacterial nanocellulose films with argon plasma at a pressure as low as 7 Pa. The ultimate pressure of this device, otherwise intended for thin-film deposition, is about 5 Pa, so the atmosphere in the processing chamber contained a significant concentration of residual gases, typically water vapor. Plasma was sustained by a glow discharge at a voltage of 680 V and a discharge current of 15 mA. The treatment times were 1, 4, and 8 min. As-synthesized bacterial cellulose pellicles were dried and subjected to plasma treatment. As deduced from XPS survey spectra, the concentrations of C and O in the surface film were 56 and 44 at.%, respectively. Plasma treatment caused a decrease in the oxygen concentration to 42 and 41 at.% for 1 and 4 min treatment, respectively. The WCA of as-synthesized and dried pellicles was 64°, and it decreased to 25 and 33° for samples treated with plasma for 1 and 4 min, respectively. The surface-to-mass ratio after plasma treatment increased by an order of magnitude. A rich morphology of samples treated for 8 min was observed by scanning electron microscopy (SEM). Treatment with argon plasma caused etching, which was also proved by measuring the weight of the nanocellulose films, which decreased linearly with increasing plasma treatment time. ATR-FTIR was also used for sample characterization, and only marginal modifications were observed, the same as already reported by Kusano et al. [8].

A direct current (DC) glow discharge was also applied to modify bacterial nanocellulose by Stankova et al. [37]. Argon plasma was sustained at a pressure of 7 Pa and voltage of 750 V, and the selected treatment time was 4 min. The ultimate pressure of the plasma device was about 5 Pa. Purified hydrogels were solidified by air-drying at ambient conditions or by lyophilization. The WCA was measured on air-dried samples, and the plasma treatment caused hydrophilization since the WCA dropped from 65 to about 40°. Rapid hydrophobic recovery was observed because the WCA approached the original value of untreated samples after storing the as-synthesized dry bacterial nanocellulose films in ambient conditions. The lyophilized films were super-hydrophilic since a water droplet soaked almost immediately after deposition. Plasma treatment of the bacterial nanocellulose films was beneficial for the adhesion and proliferation of normal human dermal fibroblast cells.

Zywicka et al. [38] treated bacterial nanocellulose films with argon plasma. The bacterial nanocellulose pellicles were homogenized to obtain pulp, which was poured into Petri dishes. The pulp was frozen and lyophilized to obtain dry sponge-like films. The films were exposed to low-pressure argon plasma sustained by a capacitively coupled radio frequency (RF) discharge operating at 40 kHz. The gas pressure was 60 Pa, and the treatment times varied between 1 and 30 min. The discharge chamber had a volume of ~2 L, and the maximum power was 100 W, so the power density was as low as 0.5 W cm^−3^. ATR-FTIR was used to detect modifications obtained on the dry sponge-like films after plasma treatment. As with other authors, the differences in the ATR-FTIR spectra were not pronounced, except at 1720 cm^−1^. Based on the measured ATR-FTIR spectra, the authors concluded that the treatment with argon plasma may lead to the formation of aldehydes and carboxylic acids, which they confirmed by the presence of auto-bands at 1720 cm^−1^. The authors also found increased concentration of the carbonyl groups. The C–O–C asymmetric stretching and C–O stretching vibrations of glycosidic bonds of the cellulose backbone did not change, so the authors concluded that the plasma treatment did not result in significant degradation or depolymerization of their material. However, prolonged plasma treatment caused brittleness of the dry sponge-like films, so the authors found 10 min to be the optimal treatment time. The characterization was repeated some months after the plasma treatment, and the spectra did not change significantly, so the authors concluded that the plasma-induced modification was permanent. X-ray diffraction (XRD) showed that the crystallinity of the bacterial cellulose slightly decreased after prolonged plasma treatment, which might result from the radiation damage caused by bombardment with Ar ions. The materials were found to be useful for respiratory masks.

**Figure 6:**
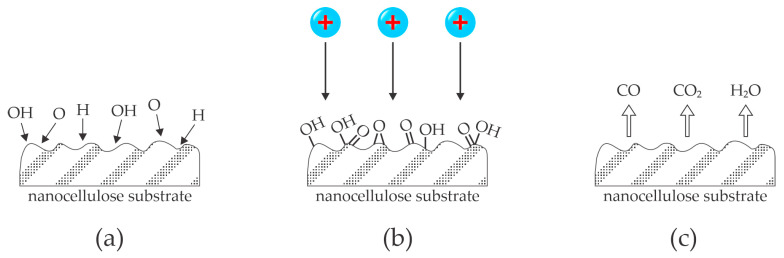
Illustration of the etching of cellulose samples upon exposure to low-pressure plasma sustained in a noble gas and residual atmosphere. (**a**) Water vapor is efficiently dissociated in such plasma, and the radicals move randomly in the gas phase and reach the cellulose surface; (**b**) the radicals interact chemically and form a functionalized highly wettable cellulose, which is bombarded with positive ions; (**c**) the ions supply enough energy to cause rapid desorption and thus cellulose etching and loss of surface functional groups.

Low-pressure plasmas suitable for treating polymer materials such as nanocellulose products are characterized by a low power density and the ability to treat samples with energetic ions. Kutova et al. [36], Stankova et al. [37], and Zywicka et al. [38] used argon as plasma gas. The residual atmosphere in these vacuum systems is often water vapor, which desorbs from the reactor’s surfaces. The cellulose samples may also represent a source of water vapor. The dissociation energy of water vapor is much lower than the excitation energy of Ar metastables, let alone Ar ionization energy, so the available power is spent on the formation of OH, O, and H radicals. These radicals interact chemically with polymer surfaces, which should enable significant cellulose functionalization and, thus, increased wettability. The reported WCAs, however, were only moderate. Furthermore, Kutova et al. [36] reported increased WCA with increasing treatment time. The paradox is explained by the combined effect of chemically reactive species and argon ions. The samples are bombarded with Ar^+^ ions, which enrich the surface reactions and may cause reactive ion etching, so the functionalized surface film is removed. The interaction between low-pressure argon plasma and nanocellulose foils is illustrated in Figure 6.

### 3.3. Treatment of Powder Dispersed in Liquids

**Figure 7:**
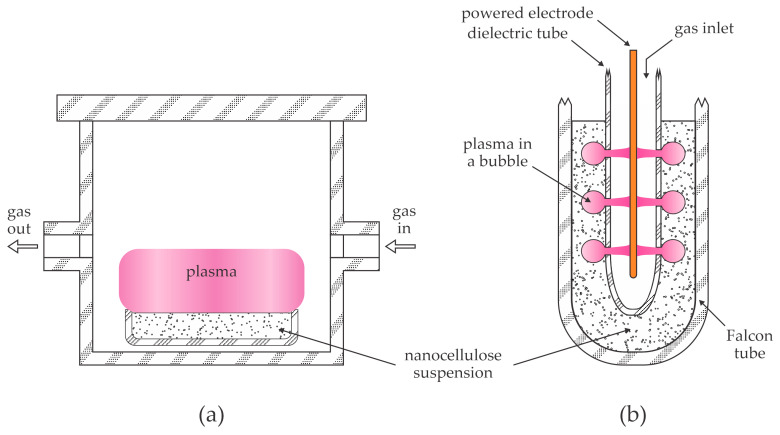
Illustration of two methods for treating water suspension of nanocellulose: (**a**) sustaining plasma of a large volume above the liquid; (**b**) bubbling plasma into the liquid.

The treatment of dry nanocellulose powder is impractical because it should be thoroughly dried to prevent aggregation of nanofibers or nanocrystals. As explained in the scientific literature surveyed in Section 3.2, the casting of the nanocellulose suspension and drying usually leads to the formation of a thin film rather than powder. An alternative to the treatment of dry nanocellulose is the application of gaseous plasma to treat the nanocellulose suspension. This method is illustrated in Figure 7.

Figure 7a illustrates the application of gaseous plasma in contact with the liquid. A dish containing the nanocellulose suspension is mounted into a chamber that is filled with the selected gas, and plasma is ignited. The contact area between the suspension and gaseous plasma should be as large as possible to speed up the process. As shown in Figure 7a, plasma in the chamber could be sustained either at atmospheric or low pressure. The pressure in the chamber should be above the saturated vapor pressure of the liquid at a selected temperature; the vapor pressure increases with increasing temperature. Nanocellulose is usually suspended in water. The water vapor saturated pressure is roughly 3000 Pa at room temperature and about 500 Pa at 0 °C. Any attempt to decrease the pressure in the chamber shown in Figure 7a will cause extensive water evaporation, but the pressure will not drop below the saturated water vapor pressure. Prolonged pumping of the chamber will cause cooling of the suspension, eventually freezing the suspension, thus making the plasma treatment inappropriate. A water solution of nanocellulose in a Petri dish thus cannot be treated at pressures as low as reported by Kutova et al. [36], Stankova et al. [37], and Zywicka et al. [38]. Namely, the pressures below water vapor saturated pressure are not achievable for treating nanocellulose in water but are useful for treating dry nanocellulose, which does not degas much upon vacuum conditions.

Plasma at elevated pressure is rarely inhomogeneous in a large volume and tends to shrink to a small volume. The choice of suitable discharges for treating large surfaces of the water suspension of nanocellulose, as illustrated in Figure 7a, is limited. One of the best solutions is the application of coplanar discharges, as illustrated in Figure 4. Coplanar discharges can be sustained at any pressure, including atmospheric pressure. Coplanar discharges provide uniform plasma over virtually unlimited surfaces and provide dense plasmas, as explained by Vida et al. [31]. The gap between the dielectric plate with the electrodes and the liquid should be about 1 mm, as illustrated in Figure 4.

An alternative to the configuration shown in Figure 7a is bubbling a selected gas through the water suspension, which is illustrated in Figure 7b. The liquid is in a suitable container, often a Falcon tube. A dielectric tube with one or several holes is immersed in the water suspension. The tube is pressurized slightly above the atmospheric pressure to enable the formation of bubbles in the holes. An electrode is immersed into the dielectric tube and connected to a suitable high-voltage, high-impedance power supply. Plasma is sustained in the bubbles, so plasma species treat the liquid. Bubbles may detach from the holes, but the constant supply of the processing gas enables the continuous formation of the bubbles. The choice of gas is not particularly limited, but the contact with the water solution always enables the presence of water vapor in the bubbles. The partial pressure of water vapor cannot be larger than the saturated vapor pressure at the selected temperature. Direct contact of the water solution with the electrode is discouraged because the available power will be spent on ohmic heating rather than sustaining gaseous plasma because of the final electrical conductivity of the suspension.

Panaitescu et al. [39] proposed a plasma treatment of the water suspension of bacterial nanocellulose with plasma sources attached to the suspension. Plasmas were sustained either in pure argon or an admixture with a reactive gas (oxygen, nitrogen, or ammonia). The discharge power was about 100 W, and the treatment time was half an hour. The authors used the configuration shown in Figure 7a, but plasma was sustained in a filamentary jet or a plasma torch, so the area of the interface between plasma and water suspension was only about 1 cm^2^. The suspension was then deposited on silicon substrates, dried, and probed using various techniques. The authors concluded that the plasma treatment of the water solution of bacterial nanocellulose caused cross-linking as well as minor changes in the oxygen concentration as determined by XPS. The pristine nanocellulose contained 35 at.% oxygen, and the concentration was between 33 and 37 at.% after treating with plasmas sustained in argon with different admixtures of reactive gases. The authors concluded that the plasma treatment reduced the concentration of low-molecular-weight impurities and thus caused the purification of the bacterial nanocellulose.

Chiulan et al. [40] treated a water suspension of cellulose nanofibers by immersion of a plasma torch into the suspension. They used the configuration shown in Figure 7b but with only one hole in the dielectric tube. Plasma was sustained in argon, which was blowing continuously through the discharge tube into the water suspension. The discharge power was as large as 150 W, and the treatment time was half an hour. The plasma-treated suspension was freeze-dried in order to form sponges. The sponges synthesized from the untreated suspension contained relatively large pores and agglomerated fibers, while those synthesized from plasma-treated suspension showed more individual nanofibers forming small bundles. The authors concluded that the plasma treatment led to defibrillation and suppressed agglomeration of the nanofibers. Plasma treatment also caused fiber breaking. The sponges were compressed in order to form films. The wettability of the films was measured. The compressed sponges synthesized from untreated nanofibers exhibited a super-hydrophilic surface finish. Namely, the water contact angle was impossible to measure because the deposited water droplet was absorbed instantly. The WCA on the plasma-treated compressed sponges was about 11°. The plasma treatment thus caused weak hydrophobization, which the authors explained by a decreased amount of the secondary alcoholic groups. Poly(ethylene glycol)methyl ether methacrylate was added to the suspension for grafting the cellulose nanofibers with a hydrophobic coating. A significant difference between untreated and plasma-treated suspension was reported. Namely, the compressed sponges containing grafted nanofibers without plasma treatment exhibited a WCA of 14°, while for those with plasma treatment, the WCA was 42°. The authors explained the more hydrophobic character of the cellulose fibers and thus higher WCA of grafted biners by carbonyl groups on the cellulose surface after grafting. Also, the plasma treatment and grafting led to a decrease in the degree of crystallinity of the cellulose nanofibers. The authors also reported that plasma treatment favored the release of bound water.

## 4. Conclusions

**Figure 8:**
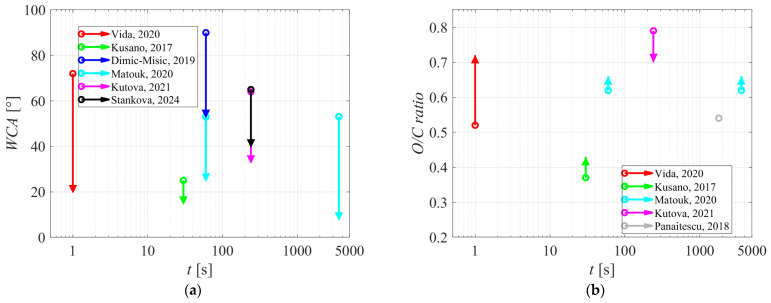
(**a**) The initial and final water contact angles; (**b**) oxygen-to-carbon ratios versus the plasma treatment as reported by different authors. The following plasmas were used: coplanar DBD, air, 1 bar [31]; DBD, helium, 1 bar [8]; DBD, nitrogen, 1 bar [32]; DBD, argon + ammonia, 1 bar [35]; DC, argon, 7 Pa [36]; DC, argon 7 Pa [37]; treatment of water suspension [39].

The science of plasma treatment of nanocellulose is still in its infancy. One obstacle that is demonstrated from the reviewed scientific literature is a significant discrepancy in the surface wettability of as-synthesized materials before the plasma treatment. Some authors reported a super-hydrophilic surface finish of as-synthesized nanocellulose, but others found measurable water contact angles. The WCA measured for the as-synthesized nanocellulose ranged between 0 and 90°. The plasma treatment usually causes increased wettability, but the results reported by different authors are highly scattered, so any conclusive observations were challenging. Still, for the sake of completeness, the initial WCA and the WCA after plasma treatment are shown in Figure 8a. Many authors have reported the oxygen concentration in the surface film probed by XPS. Figure 8b reveals the initial ratio between oxygen and carbon concentrations and the final ratio after the plasma treatment. Both the change in surface wettability and the [O]/[C] are plotted versus the reported treatment time.

Another reason for large discrepancies between the results reported by different authors is the application of different plasmas sustained at different pressures by different discharges. As stressed in Section 2 of this review, the surface finish (composition, wettability) depends on the dose of radicals. Unfortunately, no author reported the fluxes of plasma species. Still, plasma treatment caused modifications of the surface properties of nanocellulose, so the reviewed papers could be regarded as pioneering ones in the niche with vast potential for applications.
